# Microfabricated tuneable and transferable porous PDMS membranes for Organs-on-Chips

**DOI:** 10.1038/s41598-018-31912-6

**Published:** 2018-09-10

**Authors:** W. F. Quirós-Solano, N. Gaio, O. M. J. A. Stassen, Y. B. Arik, C. Silvestri, N. C. A. Van Engeland, A. Van der Meer, R. Passier, C. M. Sahlgren, C. V. C. Bouten, A. van den Berg, R. Dekker, P. M. Sarro

**Affiliations:** 1 Delft University of Technology, Department of Microelectronics, Electronic Components, Technology and Materials (ECTM), Delft, 2628 CD The Netherlands; 2 BIOND Solutions B.V., Delft, 2628 CD The Netherlands; 3 Eindhoven University of Technology, Department of Biomedical Engineering, Soft Tissue Engineering and Mechanobiology (STEM), Eindhoven, 5600 MB The Netherlands; 4 University of Twente, Applied Stem Cell Technologies, MIRA Institute for Biomedical Technology and Technical Medicine, Enschede, 7500 AE The Netherlands; 5 University of Twente, BIOS Lab on a Chip group, MIRA and MESA, Institute for Nanotechnology, Enschede, 7500 AE The Netherlands; 6 Abo Akademi University, Faculty of Science and Engineering, Molecular Biosciences, Turku, FI-20500 Finland; 7 Eindhoven University of Technology, Institute for Complex Molecular Systems (ICMS), Eindhoven, 5600 MB The Netherlands; 8 Phillips, Philips Research, Eindhoven, 5656 AE The Netherlands

## Abstract

We present a novel and highly reproducible process to fabricate transferable porous PDMS membranes for PDMS-based Organs-on-Chips (OOCs) using microelectromechanical systems (MEMS) fabrication technologies. Porous PDMS membranes with pore sizes down to 2.0 *μ*m in diameter and a wide porosity range (2–65%) can be fabricated. To overcome issues normally faced when using replica moulding and extend the applicability to most OOCs and improve their scalability and reproducibility, the process includes a sacrificial layer to easily transfer the membranes from a silicon carrier to any PDMS-based OOC. The highly reliable fabrication and transfer method does not need of manual handling to define the pore features (size, distribution), allowing very thin (<10 *μ*m) functional membranes to be transferred at chip level with a high success rate (85%). The viability of cell culturing on the porous membranes was assessed by culturing two different cell types on transferred membranes in two different OOCs. Human umbilical endothelial cells (HUVEC) and MDA-MB-231 (MDA) cells were successfully cultured confirming the viability of cell culturing and the biocompatibility of the membranes. The results demonstrate the potential of controlling the porous membrane features to study cell mechanisms such as transmigrations, monolayer formation, and barrier function. The high control over the membrane characteristics might consequently allow to intentionally trigger or prevent certain cellular responses or mechanisms when studying human physiology and pathology using OOCs.

## Introduction

Lab-on-chip (LOC) and particularly Organs-on-Chips (OOCs) generally consist of a 3D Polydimethylsiloxane (PDMS)-based microfluidic structures fabricated using soft lithography^1–3^. These chips comprise a top and bottom thick moulded PDMS substrate and host microfluidic channels that are often interfaced through a porous membrane. Depending on the envisioned application, the membrane might function as co-culture support, artificial barrier or filter^4,5^. Such a membrane is normally required to have micron pore sizes and thicknesses to mimic closer topography and mechanical conditions of the human body^5^.

Commercially available membranes made of materials such as polycarbonate (PC) and Polyethylene terephthalate (PET) have been traditionally used to create such porous interface for PDMS-based OOCs^6–8^, as they are easily accessible and known to promote cell adhesion and growth. The porous surface of these materials is generally obtained by a track-etching process, using either chemical etching or ion bombardment^9^. More recently, efforts in tissue engineering have also enabled the use of electrospun materials creating highly porous biomaterials, namely nanofibrous membranes resembling scaffold-like structures^10,11^. All these materials have been lately used to study cancer metastasis, to recreate organ-capillary interfaces, cell diferentiation and proliferation among other applications^12–15^.

Nevertheless, other studies might need to precisely define the pores position and distribution to have higher control over the variables mostly influencing the mechanism under study. The contact area has been suggested to play an important role when studying notch signalling. For instance, reducing pore size with fixed spacing might affect cell-cell signaling area, with possible effects on cell fate^16^. Cell morphology and adhesion are also suggested to be influenced by the anisotropy of the membrane topology^17–19^. With track etched and electrospun membranes the control over pores positioning is cumbersome and limits their application in studies investigating the role of surface topology on cell-cell interaction and morphology. The limited transparency, in the case of PC and PET, might also interfere with optical characterization of cell responses^20^.

Alternatively, porous membranes made of parylene^21^, SU8^22^ and PDMS^23,24^ have been developed using conventional microfabrication techniques, such as photolithography and dry etching^25,26^. Unlike track etching process and electrospun deposition, such techniques allow precise positioning of the pores, providing higher accuracy and local control of the porosity and enabling the fabrication of larger-area membranes in a timely and cost-effective manner. However, including such non-conventional materials in standard microfabrication processes is not trivial and the related technological development is not as far developed as for rigid materials such as silicon, oxides, nitrides and metals. Patterning polymeric materials with features smaller than 5 *μ*m in a reproducible and reliable way is still cumbersome. Recently, Kim *et al*. optimized the lithography and etching process for parylene, fabricating porous membranes with pore sizes down to 1 *μ*m and porosity up to 40%^21^. Esch *et al*. did a similar work with SU8, reaching minimum features of 8 *μ*m for membranes down to 0.5 *μ*m thick^22^.

Despite the outstanding features achieved by the aforementioned literature and the proof of concepts developed using various materials, most of these membranes are not fully suited for OOCs with specific mechanical requisites, such as low stiffness and elasticity, required to enable stimulation of cells and tissues through mechanical stretching. Thus, most OOCs rely on PDMS due to its well-known elasticity (ε > 5%), low stiffness (E _<_ 5 MPa) and well known biocompatibility^2–5,27^. However, patterning such polymer with standard lithography is still difficult due to its surface chemistry and thermomechanical properties^27^. On one side, previous works have focused on improving the patterning of the polymer by tuning the lithographic steps and etching conditions, successfully reducing the minimum feature size down to 4 *μ*m^23,28^. Nevertheless, the treatment of the surface prior to photoresist (PR) deposition is not sufficient to overcome uniformity issues caused by inactivated regions or topography variations across the substrate. Moreover, the photoresist is prone to crack during baking steps due to the high thermal expansion of PDMS, limiting the minimum feature sizes that can be patterned. Such non-uniformity on the polymer surface during processing causes low reproducibility and limits the maximum area patternable. On the other side, Wang *et al*. achieved 2 *μ*m pore sizes with an alternative solution based on the overlapping of two porous PDMS membranes^24^. Nonetheless, this approach requires the two layers to be processed separately and the quality of the resulting membrane is very dependent on the accuracy of the alignment and the manual procedures needed to overlap both layers.

Hence, porous PDMS membranes for OOC applications are mostly developed through replica moulding. By using such fabrication method, outstanding concepts of devices such as lung-on-chip and gut-on-chip have been reported^4,5^. However, membrane characteristics such as minimum pore size, thickness and porosity levels are constrained by this method. As replica moulding relies strongly on time-consuming manual procedures, creating thin porous membranes (<10 *μ*m) with just few micron pore sizes (<5 *μ*m), high porosity and uniform pore distribution requires extreme caution. Intrinsic issues such as unwanted adhesion of the material with the mould or blockage of the pore can easily compromise the structural stability of the highly fragile microstructures^5^. Additionally, there is always the risk of including batch-to-batch differences that also hurdles the yield and scalability of the device manufacturing. Thus, this fabrication method is currently limited provided that specific applications require smaller features^1,28^.

Nonetheless, studies suggest that controlling the membranes features could contribute to investigate a variety of biological phenomena. On one side, thin membranes (<10 *μ*m) might be essential in better recapitulating *in vivo* microenvironments using OOCs. For instance, in the case of Bruch’s membrane, a structure located between the retinal pigment epithelium and choroidal capillaries of the eye, a membrane few-micron thick might be necesarry to better mimmic the structure^29^. A recent study also suggests that particularly thinner membranes might help to improve cell visualization when studying endothelial cell-smooth muscle cell interaction, specifically in studying cell signaling taking place in arterial walls under hemodynamic loading^30^. On the other side, control over pore size can be fundamental to study mechanisms such as cell migration, critically involved in various physiological activities such as maintenance of homoeostasis, immune responses, angiogenesis, adipogenesis and embryogenesis^31–34^. For instance, this can be exploited to understand whether migration occurs in cancer-on-chips studying extra/intravasation, or endothelium-to-mesenchymal transition^35^. Big pore sizes (>70 *μ*m) might be also of interest specially in the study of processes such as chondrogenesis and osteogenesis^32,36^. Smaller pore sizes might be relevant in other cellular communication studies, where transmission between cells is performed in the form of soluble species, such as growth factors, cytokines and hormones interacting with cellular receptors^31,37^. Further understanding of how these interactions influence the function of tissues are highly relevant in pathophysiology, wound healing and developmental biology as well^38^. Therefore, when modelling multi-niche biological systems with OOCs, the ability of controlling the characteristics of the porous membrane to allow or restrict certain processes might contribute to provide more accurate models.

In this work, we propose a novel and reproducible process to fabricate transferable porous PDMS membranes for OOCs using microelectromechanical systems (MEMS) fabrication technologies. Firstly, with this process, a minimum pore feature size smaller than previously reported and a very high porosity can be realized^23,28,39^. Secondly, both pore size and porosity can be acurrately and locally tuned. The technology employed also offers a high control on the distribution of the pores across large surface areas. Thirdly, aiming to overcome issues brought by conventional replica moulding for fabricating OOCs and improve their scalability and reproducibility, we included in our process a sacrificial layer to easily transfer the membranes from the silicon substrate. The process does not need any risky manual handling when defining the critical features of the membrane (pore size, porosity), allowing to fabricate and transfer thinner functional PDMS porous membranes than before reported^39^. To confirm their biocompatibility two cell types; human umbilical endothelial cells (HUVEC) and MDA-MB-231 (MDA) cells were cultured on the membranes fabricated and transferred with our process. We present two brief cases to evaluate the applicability of the porous membranes. The first case focuses on MDA cell morphology and transmigration, and the second one highlights HUVECs migration and barrier function of cell monolayers.

## Results

### Microfabricated Tunable Porous PDMS Membranes

**Figure 1:**
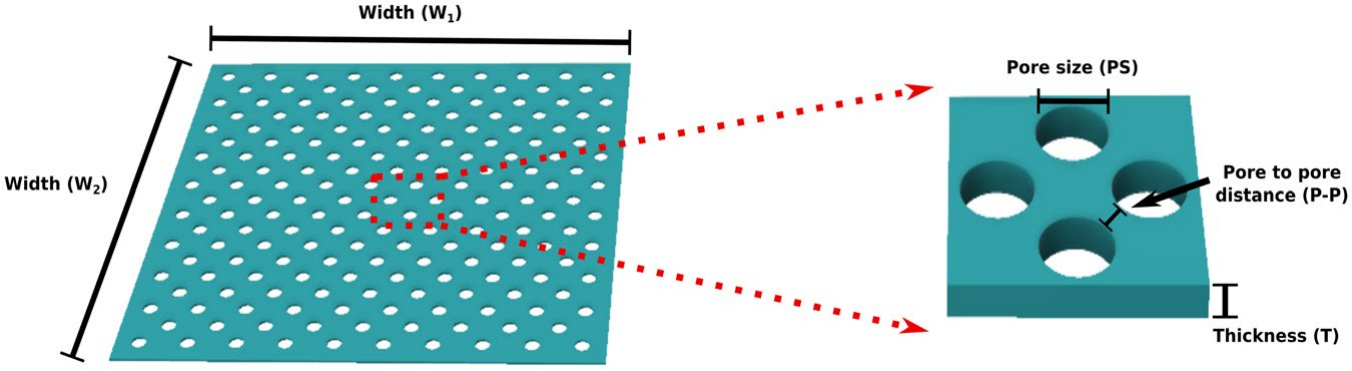
Three dimensional sketch of a porous PDMS membrane specifying the adopted terminology: pore size (*PS*), pore to pore distance (*P-P*), thickness (*T*) and area (*W*_1_ × *W*_2_).

Highly porous PDMS membranes were fabricated using conventional IC and MEMS fabrication technology in a cleanroom facility (Class 100, ISO 5). The characteristics of the membranes are considered based on parameters such as pore to pore distance (*P-P*), size (*PS*) and thickness (*T*) of the layer, as depicted in Fig. 1. The porosity, defined by the ratio between the volume of voids to the total volume, was successfully tuned by varying *PS* and *P-P* (Supplementary Eqs S1 and S2).

**Figure 2:**
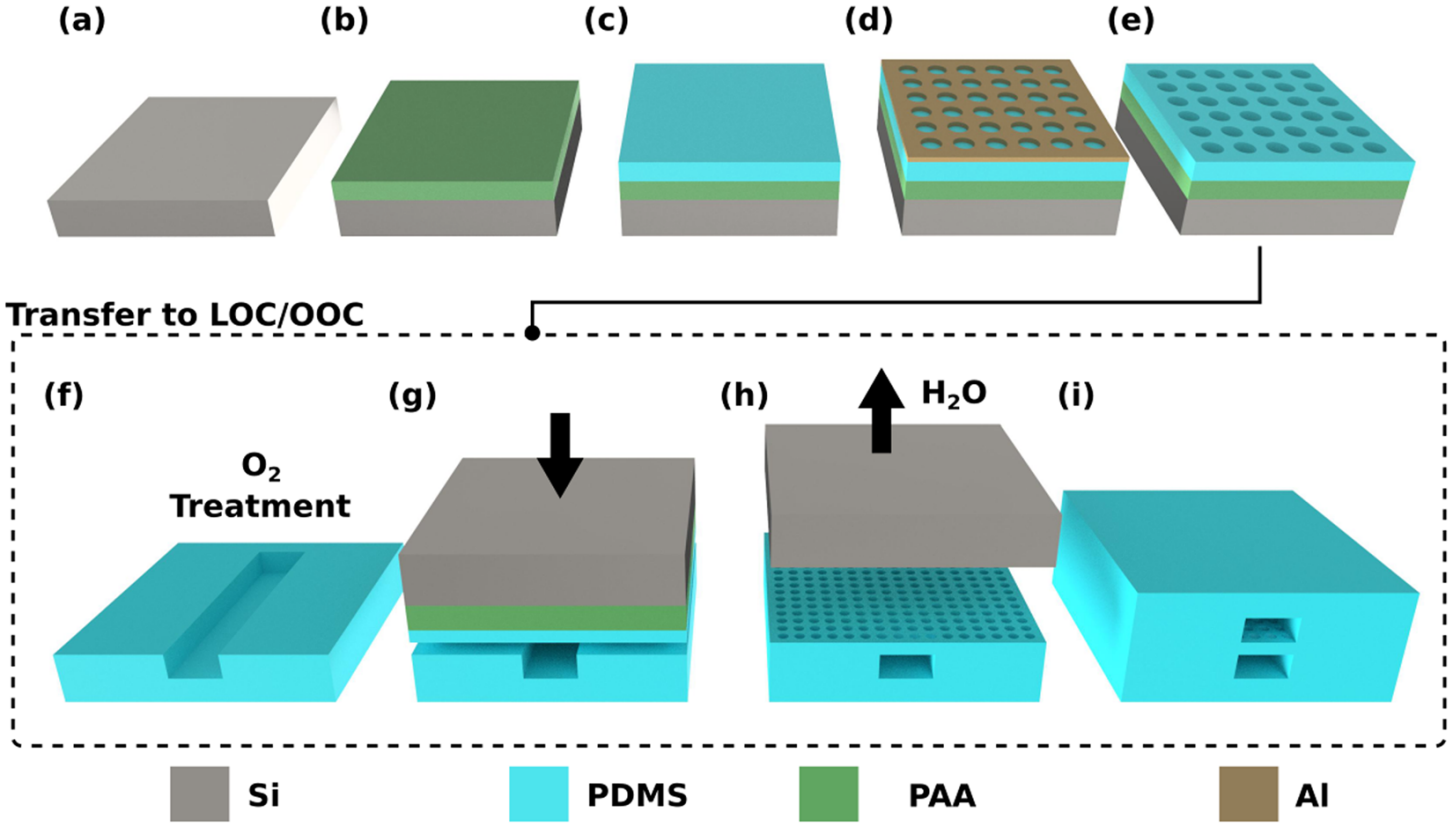
Schematic illustration of the microfabrication process (from (**a**–**e**)) and the transfer (from (**f**–**i**)) of the porous PDMS membranes to OOCs. (**a**,**b**) Deposition of the sacrificial and water-soluble poly (acrylic acid) (PAA) layer by spin coating. (**c**) Deposition of the PDMS by spin coating to define the membrane thickness. The layer is then thermally baked. (**d**) Deposition and patterning of the Al masking layer to define the desired pore features (*PS* and *P-P*). (**e**) Dry etching of the Al and the PDMS layers. The Al masking layer is then removed by wet etching, leaving exposed the patterned PDMS surface. (**f**) First step required for transferring the membrane: oxygen plasma treatment on the PDMS membrane and on the bottom surface of the PDMS-based OOCs. (**g**) The porous membranes, carried by the silicon substrate, are placed in contact with the activated surfaces of the OOCs and then kept under a constant pressure to promote mechanical bonding. (**h**) Releasing of the porous membranes by dissolving the sacrificial layer (PR or PPA)in water in an ultrasonic bath. (**i**) Final assembling of the OOC by attaching the top part to complete the microchannel top side.

**Figure 3:**
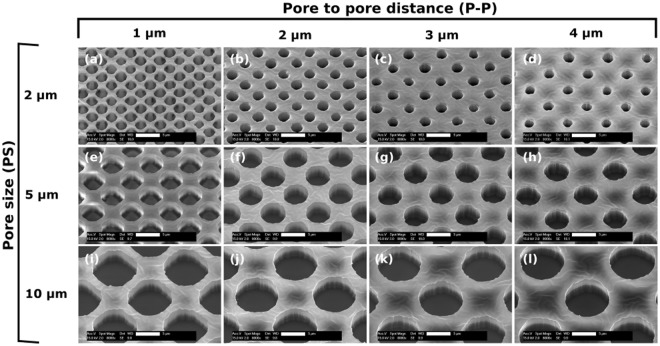
SEM images of the different patterned 4 *μ*m-thick porous PDMS layers, with various *P-P* and *PS*, taken at a fixed magnification (8000x) and tilting angle of 26°. Scale bars: 5 *μ*m.

The process, depicted in Fig. 2a–e, was tailored for achieving high control over the pore size, and porosity by tuning the thermal budget of the lithography process and by including an Aluminum (Al) masking layer to improve both the Photoresist (PR) to PDMS adhesion and the mechanical stability. In fact, the use of an Al mask guarantees a highly uniform PR layer crucial for achieving small pore sizes. Substrates layers without significant structural defects were obtained at wafer level, corresponding to porous surface areas as large as 78 cm^2^. The developed lithographic process allowed to microfabricate membranes with *PS* from 2.0 ± 0.3 *μ*m to 10 ± 0.3 *μ*m and *P-P* from 1 *μ*m to 4 *μ*m (Fig. 3). The minimum *PS* achieved, 2.0 ± 0.3 *μ*m (Fig. 3a), is two times smaller than previously reported^24^.

In Fig. 3 scanning electron microscopy (SEM) images highlighting the wide range of porosity achieved are shown. In particular, the porosity ranges from 8% to 65%, demonstrating a significantly extended range compared to what reported by others^22–24^. The highest porosity (65%) corresponds to the layers with *PS* = 10 *μ*m and *P-P* = 1 *μ*m (Fig. 3i) while the lowest (8%) corresponds to *PS* = 2 *μ*m and *P-P* = 4 *μ*m (Fig. 3d). To achieve the various porosities with photolithography, the exposure time was kept constant and the development time was properly tuned. All layers were imaged by SEM with the same magnification (8000x) and tilting angle (26°) to show the same perspective. Moreover, cross-section images of the layers were obtained during the experiments to determine the complete etch through of the PDMS and to investigate the etched wall profile. An example of those images can be found in Supplementary Fig. S1.

### Transfer of Microfabricated Porous Membranes to OOCs

**Figure 4:**
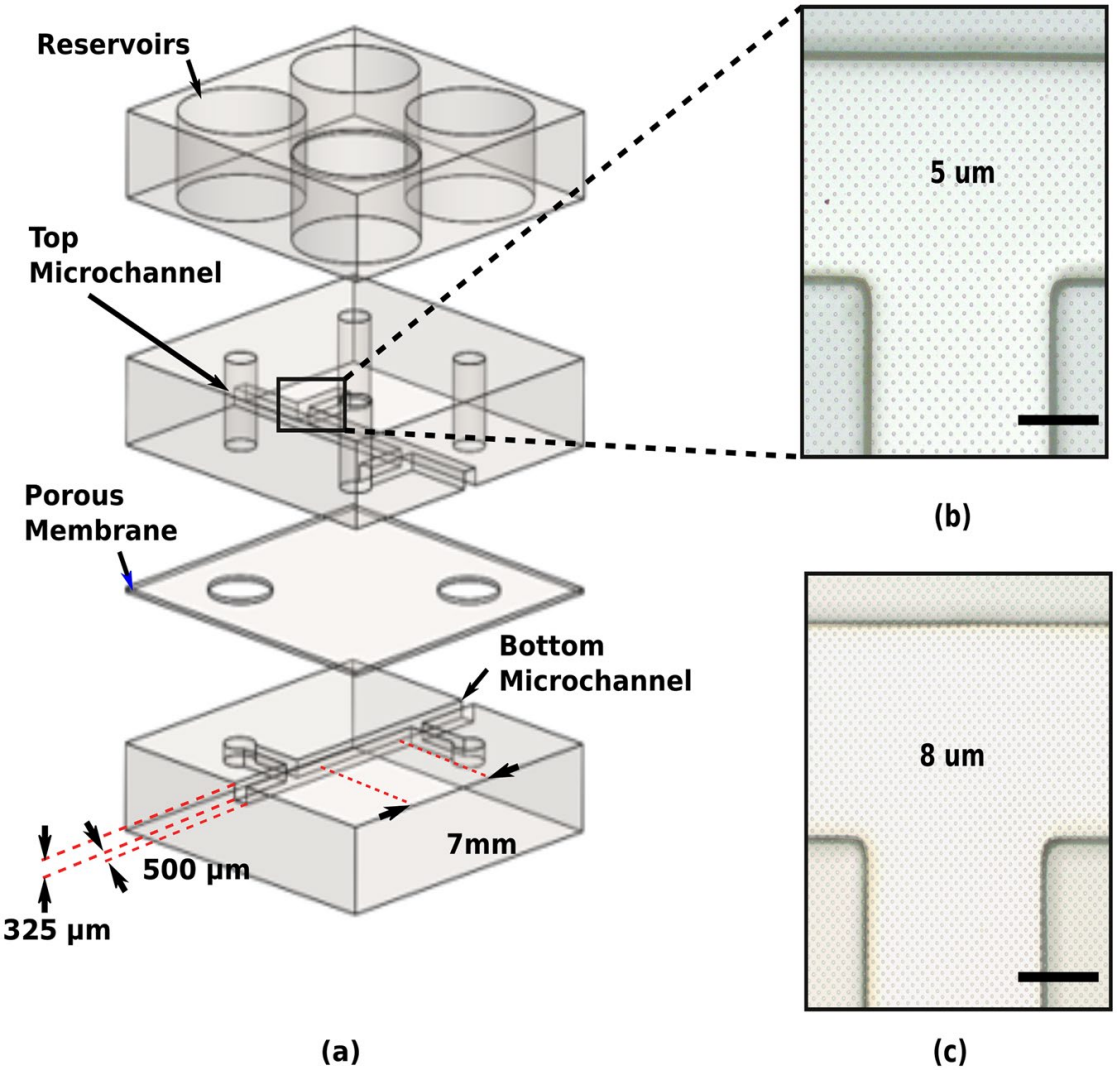
The architecture used in the experiments of HUVECs culturing on transferred PDMS membranes. (**a**) Schematic representation of the OOC. A four-layered sandwich consisting of bottom layer with a defined microchannel (500 *μ*m width, 325 *μ*m height, 7 mm in length), porous PDMS membrane with defined pores (8 *μ*m diameter, 4 *μ*m in thickness), top layer with the same channel dimensions as the bottom layer, and PDMS slab with defined reservoirs for media refreshing (5 mm in diameter). (**b**) Close-up of the microchannel area with transferred membrane of 4 *μ*m in pore size. (**c**) Close-up of the microchannel area with transferred membrane of 8 *μ*m in pore size. Scale bars: 100 *μ*m.

In combination with the advanced microfabrication process previously described, a novel method to transfer the tunable porous PDMS membranes was also developed to extend its applicability to most OOCs (Fig. 2f–i). After defining the features of the porous membranes, it is possible to transfer them to any PDMS-based OOC by using a sacrificial layer. Two materials were investigated and used as sacrificial layer, PR and poly (acrylic acid) (PAA), in order to transfer of the microfabricated membranes from the silicon substrate to two different OOCs. In preliminary experiments using PR as sacrificial layer, which has already been used to release thin non-porous PDMS membranes^40^, bigger PR residues were always observed after transferring (Supplementary Fig. S2a). These residues were detected both inside the microchannels and on the surface of the membranes. On the contrary, it has been observed that the usage of PAA as sacrificial layer facilitate the release of the porous membranes from the silicon carrier. As depicted in Fig. 2h, the PDMS assembly was only submerged in deionized (DI) water in an ultrasonic bath. After 10 min the assembly detaches from the silicon substrate. The results demonstrate that PAA guarantees a cleaner surface as well as no residues inside the microchannel (Supplementary Fig. S2b). The few residues observed in a couple of samples during the fabrication experiments were easily removed in DI water. In Fig. 4a, optical microscope images of transferred membranes, of 8 *μ*m and 4 *μ*m in pore size, are reported for one of the OOC architectures used for the biocompatibility assessments^41^.

**Figure 5:**
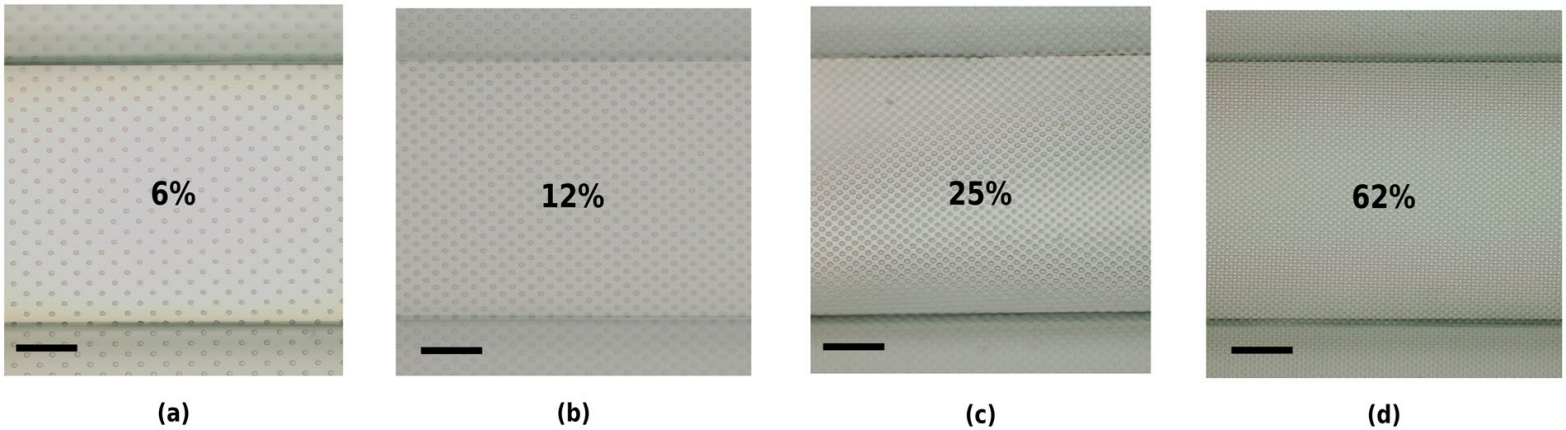
Optical images of 8 *μ*m pore size, 4 *μ*m-thick membranes of different porosities transferred to OOCs. Porosity value: (**a**) 6%, (**b**) 12%, (**c**) 25% and (**d**) 62%. Scale bars: 125 *μ*m.

Numerous transfer procedures for different porosities and *PS* were successfully performed (Fig. 5), achieving a transfer success rate higher than 85%. A transfer process is considered successful when no sagging of the membrane nor PAA residues on the microchannels are observed.

### MDA Cell Transmigration and Morphology

**Figure 6:**
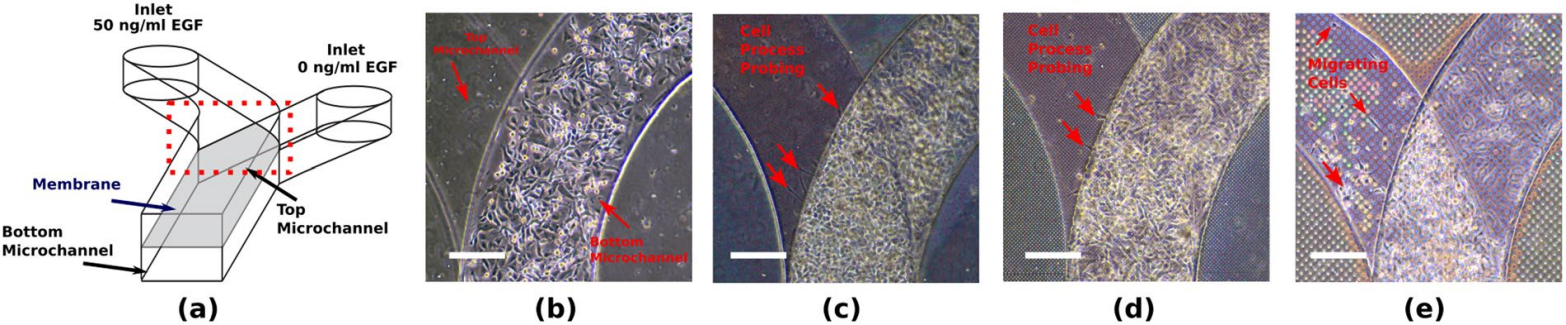
MDA cell transmigration through porous PDMS membrane transferred to a OOC. (**a**) Schematic of the OOC used for EGF driven migration, showing the inlets for top and bottom microchannel (400 *μ*m width, 100 *μ*m height) where EGF is added. Cells were seeded at the bottom side of the membrane, serum starved overnight and exposed to a gradient of EGF towards the top channel, separated with either a (**b**) nonporous; (**c**) 2.0 ± 0.3 *μ*m pore size (Type A); (**d**) 3.0 ± 0.3 *μ*m pore size (Type B) and (**e**) 10.0 ± 0.3 *μ*m pore size (Type C) membrane. Arrows indicate cells (**e**) or cellular processes (**c**,**d**) probing in the channels. Scale bar: 50 *μ*m.

Using an OOC based upon the lung-on-a-chip architecture^42^, MDA cells were cultured in two parallel microchannels separated by a transferred porous PDMS membrane to investigate transmigration (Fig. 6a). Membranes were fabricated with either 2.0 ± 0.3 *μ*m (Type A), 3.0 ± 0.3 *μ*m (Type B) or 10.0 ± 0.3 *μ*m (Type C) pore size (*PS*), with fixed porosity (40%) to investigate whether cell transmigration occurs when using such artificial barrier. MDA cells were cultured on the bottom side of the membrane and attracted with EGF administered from the top side (Fig. 6a). Cells were also cultured in a device with a non-permeable membrane as reference sample and no cell migration was observed. Type A and Type B membranes allowed for processes of cells probing the top inlet (Fig. 6c,d), but no migration into the top inlet was observed. Type C membranes allowed for transmigration and cells migrating towards the EGF gradient (Fig. 6e). To further study this transmigration across the membranes, MDA cells were labeled with different florescent colors and then seeded on either side of a membrane to evaluate exchange between the two sides after 24 hours. In the case of Type C membranes, some cells were seen to migrate completely through the membrane (Supplementary Fig. S3a–c), whereas for Type B membranes only sporadic processes were protruding through the membrane (Supplementary Fig. S3d–f).

**Figure 7:**
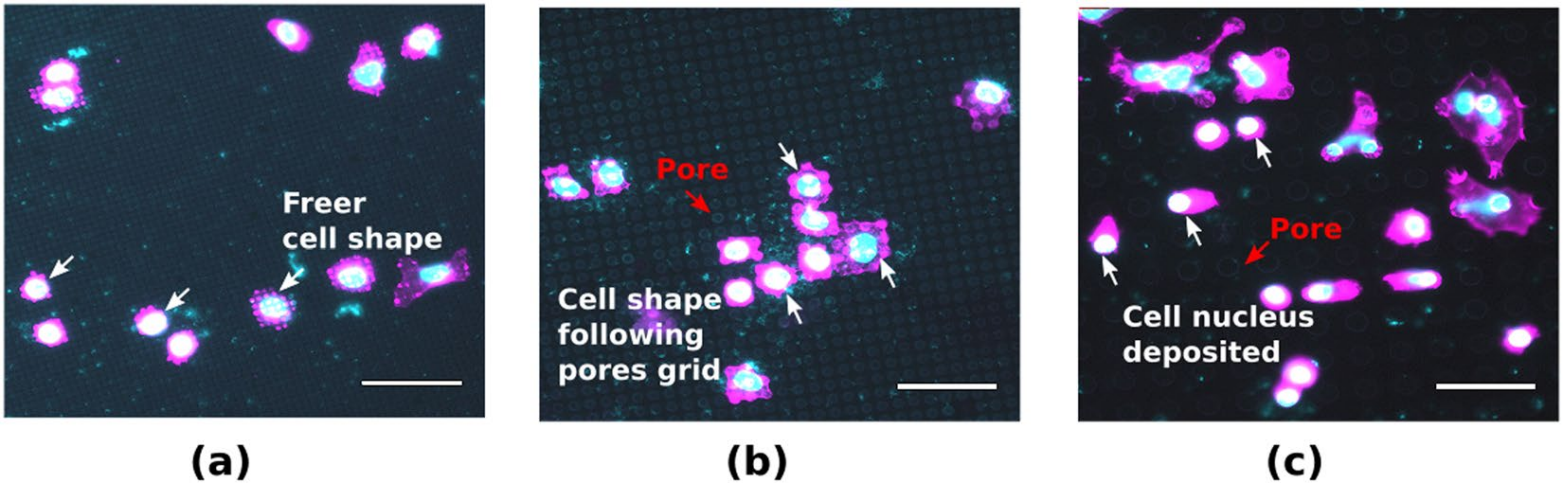
MDA interaction with porous PDMS surface. Cell and nuclear morphology was imaged on PDMS porous membranes transferred to a PDMS substrate. Pore size of the PDMS membranes (**a**) 2.0 ± 0.3 *μ*m (Type A) (**b**) 3.0 ± 0.3 *μ*m (Type B) and (**c**) 10.0 ± 0.3 *μ*m (Type C). Scale bar: 50 *μ*m.

Additionally, to briefly investigate the effect of the different pore sizes of the microfabricated membranes on cell morphology, MDA cells were also cultured on membranes transferred onto a flat PDMS substrate. In the case of Type A and Type B pores, cells grow on top of the membrane and can probe into the pores, as demonstrated by polymerized actin condensation at the pores (Fig. 7a,b). On membranes with Type C pores, cells can deposit their nucleus entirely into the pore and extend the rest of the cell body towards a neighbouring pore (Fig. 7c). Although descriptive, another remarkable difference is the effect on the shape of cells, with the cells following the grid of the pores, especially in the case of the Type B and the Type C pore membranes. This leads to rectangular or even linear shapes, whereas the Type A pores leave more freedom in cell morphology.

### HUVEC Transmigration and Barrier Integrity

**Figure 8:**
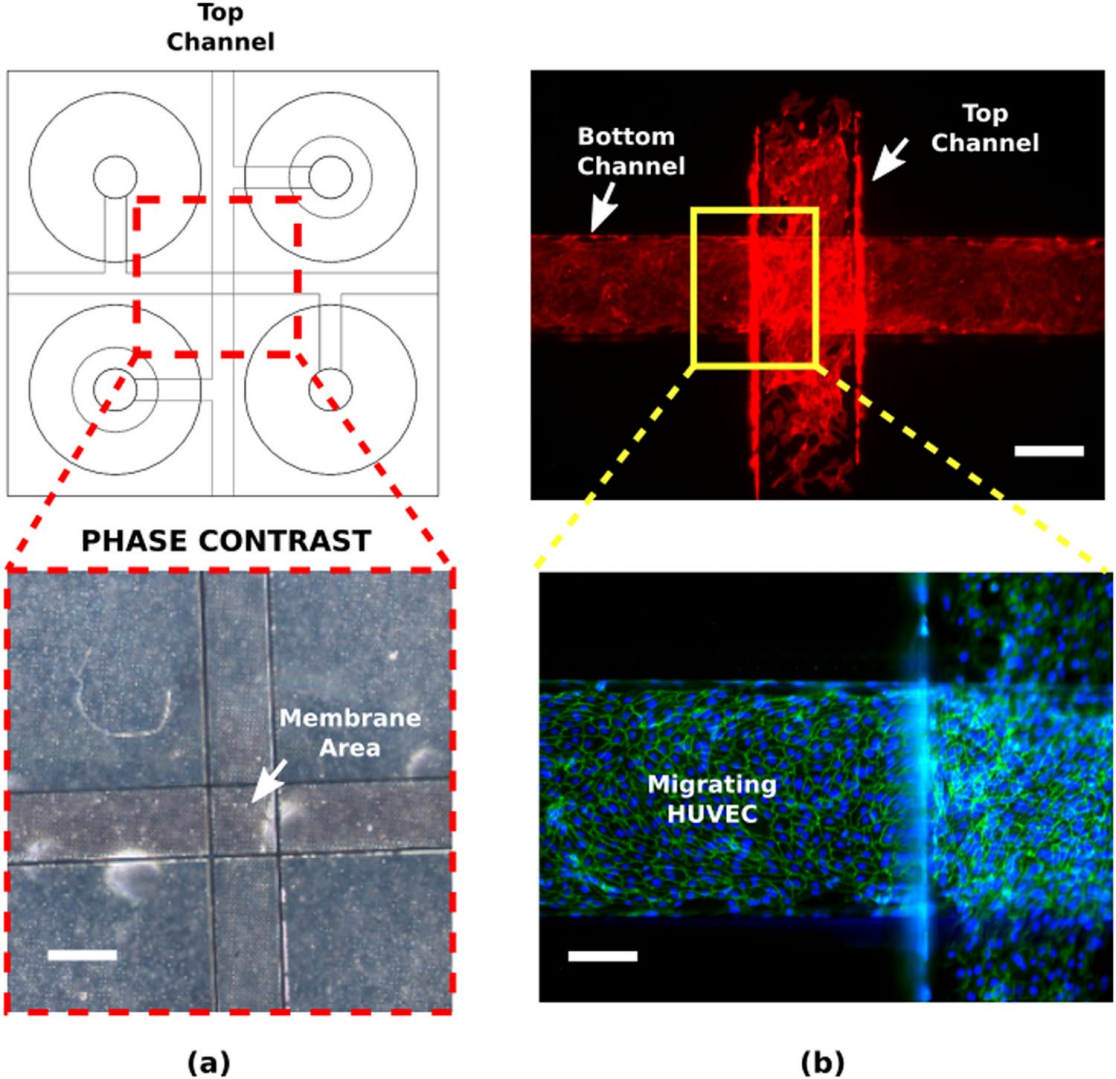
Porous PDMS membranes provide support for cell culturing. (**a**) A top view schematic of the OOC chip with a Phase contrast image of the membrane area, which is transparent giving visibility to the PDMS different layers. (**b**) Initially seeded to the top channel, cell migrated to the adjacent channel upon prolonged culturing. Staining of cell-cell junction proteins (B-bottom) indicates a healthy barrier formation by the cells. Red: phalloidin, cytoskeleton; green: VE-Cadherin, adherens junctions; blue: DAPI, nuclei. Scale bars: 100 *μ*m.

In this section cell migration experiments of HUVEC seeded on the OOC of Fig. 4 are reported. Cells were cultured on 8 ± 0.3 *μ*m pore size membranes with 25% porosity. Initially seeded only on the top channel of the device, HUVEC migrated through the membrane to the bottom channel of the device (Fig. 8). Cells formed cell-cell junctions as it can be seen from the adherens junction protein VE-Cadherin stainings (Fig. 7b, bottom), which indicates a healthy population with a well-established cellular barrier formation. We confirmed that the cell migration was not due to the seeding but governed by the cell behavior. Cell staining performed after 2 hours of seeding did not show cells under the membranes whereas, after 18 hours, cells were seen in the bottom channel (Supplementary Fig. S4).

In addition to the cell migration, we briefly examined the barrier function of endothelial monolayers based on fluorescent dye diffusion, analogous to a technique that is extensively used in ophthalmology to assess barrier integrity of blood vessels (Supplementary Fig. S5). We assessed whether the barrier formed by the porous PDMS membrane have an influence in the diffusion of the fluorescent dyes. The same OOC with transferred membranes with 8 ± 0.3 *μ*m pore size membranes and 25% porosity was used. Upon dye administration to the upper channel, dye diffusion is starting within 30 s. On the contrary, the device containing a monolayer of HUVEC exhibited no dye diffusion to the bottom channel, which demonstrates the formation of a well-established endothelial barrier on the PDMS membranes, despite the high porosity and large pore size. Upon quantification of fluorescent intensity (Supplementary Fig. S5b), the values were significantly higher for membranes without cells, as we found 140 times higher fluorescent intensity in empty devices.

## Discussion

Along the fabrication process of the porous PDMS membranes, the thermal budget is the most critical factor to be controlled during the lithography steps to achieve the reported minimum features. The temperature was kept lower than 90 °C to both prevent cracking of the photoresist due to thermal expansion mismatch, and avoid high degassing of the polymeric layers. Smaller features (*PS* < 2 *μ*m) were not possible to obtain without affecting the shape, uniformity and distribution of the pores during development steps of the photolithographic process.

Numerous experiments performed allowed to determine the optimal procedure to transfer clean and flat microfabricated porous membranes to the OOCs. Using PAA as sacrificial layer guarantees a higher reproducibility and no detachment, rupture or sagging of the membrane. Its high solubility in water makes the transferring easier and more reliable. When using photoresist, residues were always present which are possible to clean partially with a longer rinsing in methanol and acetone but unavoidably causing unwanted detachment of membranes in sporadic areas. Long-time submersion in organic solvents is well known and reported to affect the surface of PDMS causing swelling or detachment of the layers^43^, which most likely explains the observed unwanted detachments.

The process here presented can be easily adapted to bigger wafer sizes, further increasing the final porous membrane area. However, additional tuning of the lithography might be required to successfully achieve the features reported under such new conditions. Unlike other works^2^, the process allows to fabricate and transfer numerous PDMS porous membranes in one day (24 h). For example, considering an average-sized OOC (3 cm × 3 cm), by processing 5 silicon substrates (10 cm diameter) in parallel and considering the success rate reported, up to 85 membranes can be fabricated and transferred. The process, based on scalable fabrication techniques, proposes an alternative that allows to increase the yield when fabricating traditional PDMS-based OOCs. However, this process is not completely feasible for rapid and low-cost prototyping, as its implementation requires specialized facilities more suitable for higher scale manufacturing.

In this work we observed cell migration through the porous PDMS membranes with HUVEC and MDA cells. In the experiments performed with MDA cells, transmigration or protrusions were completely absent at Type A membranes and nonporous membranes, although small protrusions may be below the detection limit of the imaging setup (Supplementary Fig. S3a–d). MDA cells have been shown to be able to migrate through a 3 *μ*m wide slit opening, causing rupturing of the nuclear lamina^44^. This likely requires the unrestricted expansion of the nucleus in one dimension. However, in our experiments the absence on the transmigration was observed for 3.2 ± 0.3 *μ*m pore sizes most likely due to a complete restriction on the nucleus on all radial directions. These results preliminary suggest that also geometry might play an important role in transmigration mechanisms, though this should be confirmed in further investigations. Additionally, the results with MDA cells indicate an influence of the surface topography created by the pores on the cell behavior. A dependence on the shape of the cell with the pore size was noted during experiments with such cell type. This suggests the potential of controlling further the cell behaviour and distribution by controlling the pore size.

In the case of HUVEC experiments, cells not only established their barrier by means of cell-cell junction protein expression, but also exhibited migration towards the bottom channel through the membrane. To discard that cell seeding to devices initially result in forced migration of cells simply by passing through the pores of the membrane, the cells were fixed and stained 2 and 18 hours after seeding (Supplementary Fig. S4). It was observed that no forced transmigration of cells to the bottom channel occurs after the seeding procedure (2 hours), while cells actively migrated towards the bottom channel after 18 hours. Therefore, while providing mechanical support for healthy cell growth, PDMS membranes allow for the study of active migration of HUVEC cells, indicating the potential of using them in investigating cell migration mechanisms.

Furthermore, as cellular barriers are essential in normal physiology of organs and tissues to establish and maintain the homoeostasis and disruption of such barriers has a critical impact in many diseases (e.g. disruption of blood brain barrier in multiple sclerosis, meningitis, encephalitis, blood retinal barrier in diabetic retinopathy, macular degeneration, pulmonary air-liquid interface in pulmonary edema)^45–47^, examination of barrier integrity can be informative on the severity of diseases and on response treatment^34^. Here, we used HUVEC as an example to asses barrier integrity using an OOC with porous PDMS membranes. We administered the fluorescein to the microchannels as soon as HUVEC monolayer was confirmed by optical inspection. Difference in dye diffusion is clear between an empty device and one with cells, as the dye diffusion started within 30 s after administration of the flourescein. As porous PDMS membranes do not impose any resistance to such diffusion while providing enough mechanical support for a healthy cell monolayers, they can be used for assessment of barrier integrity of cell layers.

The results on cell transmigration, topology and barrier formation demonstrate the biocompatibility of the porous PDMS membranes. Moreover, they contribute to highlight the importance of considering the accurate control of the pore features as a design variable when developing OOCs that more closely represents the microenvironment conditions of the phenomenon of interest.

## Materials and Methods

### Materials

Polydimethylsiloxane (PDMS) was purchased from Dow Corning as a kit containing base and curing agent (Sylgard 184). Poly (acrylic acid) (PAA) with a 12% concentration was purchased from Sigma Aldrich. Positive photoresist was obtained from MicroChemicals GmbH. Chrome photolithography masks were designed in-house using Tanner L-EDIT IC Layout software and printed externally by Compugraphics International Company. Norland Optical Adhesive (NOA81) was purchased from Norland Products Inc. HUVEC and corresponding endothelial growth medium (EGM-2: EBM-2 with EGM-2 SingleQuots) were purchased from Lonza. Collagen-1 (rat tail), phosphate buffered saline (PBS), Trypsin-EDTA, Formaldehyde, as well as donkey anti-goat IgG Alexa Fluor 546, 4′, CellTracker Orange, CellTracker Green, 6-Diamidino-2-Phenylindole (DAPI), and Alexa Fluor 633 Phalloidin were purchased from ThermoFisher. Triton X-100, bovine serum albumin (BSA), (3-Aminopropyl) trietoxysilane (APTES), glutaraldehyde were purchased from Sigma Aldrich.

### Fabrication of Tunable Porous PDMS Membranes

A sacrificial polymeric layer was initially deposited on a 100 mm-Si wafer (Fig. 2a,b). Two different sacrificial layers have been tested in this work: (a) a 3 *μ*m standard PR layer (PR), (b) a 0.5 *μ*m PAA layer. The photoresist was deposited by spin coating at 2000 rpm for 30 s and baked on a proximity hotplate at 100 °C for 90 s. The PAA sacrificial layer was deposited by spin coating at 4000 rpm for 40 s and baked in a temperature controlled oven at 100 °C for 1 h. Subsequently, a PDMS layer was deposited by two-step spin coating, the first spreading step at 300 rpm and the second step at 6000 rpm (Fig. 2c). The spinning time was tuned to achieve the desired layer thickness. Values ranging from 2 to 20 *μ*m can be obtained with spinning times in the 30 to 150 s range. The polymer was cured at 90 °C for 1 h. Then, an Al layer, used as hard mask, was sputtered on the polymer surface (Fig. 2d). A 1 *μ*m photoresist layer was deposited and patterned with proximity exposure. Several pore densities were achieved by changing the arrangement of the holes in the mask layout. The Al masking layer is then removed by reactive-ion etching with a Cl^−^-based plasma chemistry. Subsequently, the PDMS is etched by reactive-ion etching (Gases: CH_4_:SF_6_:O_2_:1:2:1, P: 20 mTorr, RIE Bias: 20W, ICP Power: 500W) in an ICP plasma etcher. The etching conditions were optimized to obtain anisotropic etching, so to accurately control shape and size of the pores. Finally, the Al hard mask was removed by wet etching using a buffered solution of acetic acid, nitric acid and hydrofluoric acid (Fig. 2e).

### Transfer of Porous PDMS Membranes

Once the porous membrane was patterned as previously described, the silicon substrate was diced with an automatic dicing saw to match the dimensions of the OOC. Subsequently, the device bottom part and the porous layers were treated with oxygen plasma to activate the surface and guarantee their mechanical bonding. The porous layer and the OOC bottom substrate were brought together. A minimum constant force is then applied for 8 hours on the assembly to promote the bonding between the two elements (Fig. 2f,g). Finally, the silicon substrate was detached from the PDMS chip (Fig. 2h) by submerging the PDMS and silicon assembled chip in water in an ultrasonic bath for 10 min, in the case of PAA as sacrificial layer. This procedure provided a simple transferring of the porous layers with minimum manual handling. On the contrary, when using photoresist as sacrificial layer, the bonded porous layers and silicon chip were submerged in methanol and acetone for a longer time (>20 min) to strip the sacrificial layer. An external force was applied to accelerate the stripping and to completely release the PDMS assembly from the silicon carrier substrate. This force was applied by hand by slightly bending the OOC to allow access of the stripping solvent.

### Fabrication and Assembly of the OOC

PDMS was prepared by mixing polymer base and curing agent in a 10:1 ratio (w/w). The mixture was then degassed and was poured onto a SU-8 patterned wafer and cure for 4 h at 60 °C. After that, the cured polymer was peeled from the wafer and cut into the top and bottom part of the OOC (Fig. 2f), and four inlets (1.2 mm in diameter) were punched into the top parts. Another PDMS slab was cut into chip-sized pieces and four reservoirs were punched (5 mm in diameter) (Fig. 4a).

Porous membranes were transferred as previously described. The remaining device half (top) was attached using either PDMS/toluene mortar (5:3 w/w) or plasma induced bonding (Fig. 2i). In short, for the former case, the mortar was spin coated on to a cover slip (1500 rpm, 60 s, 1000 rpm s^−1^) and a thin layer was transferred to one of the halves using an ink roller^8,48^. After aligning two halves and the additional slab with the four reservoirs, the assembled devices were baked overnight at 60 °C. In the case of bonding through plasma activation, both the top and bottom part were exposed to an oxygen plasma (Gases: O_2_, P: 20 mTorr, RIE Bias: 20 W, Time: 20 s) and brought together aligning them assisted by an optical microscope.

### MDA-MB-231 culturing

MDA-MB-231 cells (MDA) were cultured on porous PDMS membranes transferred to an OOC where the membrane served as interface separating the top and bottom microchannel. MDA-MB-231 cells (MDA) were maintained in MDA-medium (RPMI, 10% FBS, 100 U/mL Penicillin/Streptomycin) at 37 °C in humidified air with a 5% CO_2_ concentration. For all OOC experiments with MDA, the OOCs with parallel culture microchannels were first sterilized with 70% ethanol, washed with phosphate buffered saline (PBS) and coated with fibronectin and again washed with PBS. To test permissiveness of the porous membranes to cell transmigration, cells were seeded at a density of 5 × 10^4^ cells cm^−2^ on the membrane in the bottom channel. After seeding, the cells were starved overnight by loading the OOC with serum free medium. To induce migration, a diference of 50 ng mL^−1^ of Epidermal growth factor (EGF) was established between the top microchannel inlet and the bottom channel inlet, and cells were cultured for 24 hours and visualized by a phase-contrast microscope (EVOS).

To test cell exchange between the top and bottom of the porous PDMS membrane due to migration, cells were loaded with CellTracker Orange or CellTracker Green, and seeded at a density of 5 × 10^4^ cells cm^−2^ in the bottom or the top microchannel respectively. After 24 hours the devices were imaged with a Zeiss LSM510 META NLO, using a long-distance objective (LD Achroplan).

To study cell interaction with the porous surface of the PDMS membranes, they were transferred and subsequently coated with fibronectin. MDA cells were seeded at a density of 1 × 10^4^ cells cm^−2^ and fixed with 4% paraformaldehyde (PFA) the following day. Cells were permeabilized and blocked with NET-Gel (50 mM Tris, pH 7.5, 150 mM NaCl, 0.1% v/v Nonidet P40, 1 mM EDTA, 0.25% w/v Gelatin), and subsequently stained with phalloidin-alexa-488 and 4′,6-diamidino-2phenylindole (DAPI) and imaged with an epifluorescent microscope (Zeiss Axiovert 200 M).

### Surface Functionalization and HUVEC Culturing

The surfaces of the microchannels were functionalized with APTEs and glutaraldehyde. First, chips were subjected to air plasma (50 W) for 40 s (Cute, Femto Science). Afterwards 3% (v/v) APTES mixed in ultrapure H_2_O (ELGA) was added into the channels and incubated at room temperature (RT) for 5 min. Following APTEs coating, the chips were rinsed thoroughly with 100% ethanol, and incubated for 5 min to eliminate the remaining APTES. Then 10% glutaraldehyde was added to the channels, and the chips were incubated for 5 min at RT. This was followed by thorough rinsing with distilled H_2_O and drying overnight at 60 °C.

Prior to cell seeding, the microfluidic chips were rinsed with PBS and coated with 0.1 mg mL^−1^ collagen I for 30 min at 37 °C. After coating, channels were flushed with cell medium to remove non-bound collagen. First, HUVEC were obtained from a confluent flask using 0.05% Trypsin-EDTA suspended in fresh EGM-2 at Trypsin-EDTA suspended in fresh EGM-2 at 2 × 10^6^ or 5 × 10^6^ cells mL^−1^ and pipetted into the top channel of a assembled OOC. Cells were attached by incubating the chips for 30 min statically. After that, non-attached cells were washed away by flushing the microchannels with fresh EGM-2. A droplet of EGM-2 was left on the reservoirs to prevent drying. Cells were kept in static culture conditions and medium in the channels was refreshed twice daily by pipetting fresh EGM-2 into the channels.

HUVEC were cultured with EGM-2 in t175 culture flasks, coated with 0.1 mg mL^−1^ collagen I. The cells were incubated at 37 °C in humidified air with 5% CO_2_. When cells had grown to confluent monolayers, they were either used for expermients or subcultured.

## Electronic supplementary material

Electronic Supplementary Information

## Data Availability

All data generated or analysed during this study are included in this published article (and its Supplementary Information Files).
