# Supplementary material for: Microfabricated tuneable and transferable porous PDMS membranes for Organs-on-Chips
Source: Sci Rep. 2018 Sep 10;8:13524. doi: 10.1038/s41598-018-31912-6 (PMC6131253; doi:10.1038/s41598-018-31912-6)
Supplement: Supplementary file 1 — Electronic Supplementary Information [file 41598_2018_31912_MOESM1_ESM.pdf]

Microfabricated tuneable and transferable porous PDMS membranes for Organs-on-Chips

W.F. Quiros-Solano, N. Gaio, O.M.J.A. Stassen, Y.B. Arik, C. Silvestri, N.C.A. Van Engeland, A. Van der Meer, R. Passier, C.M. Sahlgren, C.V.C. Bouten, A. van den Berg, R.Dekker and P.M. Sarro.

1. Additional information on membrane porosity and microfabrication.

Table S1: Features of diverse 4um-thick porous PDMS membranes successfully fabricated and transferred with the process reported.

≈Porosity* (%)		PORE TO PORE DISTANCE (P-P)			
		1 µm	2 µm	3 µm	4 µm
PORE SIZE (PS) (µm)	2 µm	35	19	12	8
	5 µm	54	40	31	24
	10 µm	65	54	46	40

*The porosity is defined as the percentage given by the ratio between the total volume etched (void volume) divided by the total volume of the patterned layer.

Considering the diameter or pore size (PS), width (W1) and length (W2) of the total area covered by the porous pattern, thickness of the layer (T) and the pore to pore distance (P-P), the porosity can be calculated as following:

$$\text{Porosity }(\%)=\frac{\text{Void Volume}}{\text{Total Volume}}\cdot 100=\frac{\frac{\pi\cdot\left(\frac{PS}{2}\right)^{2}\cdot W1\cdot W2\cdot T}{(PS+P\text{-}P)^{2}}}{W1\cdot W2\cdot T} \qquad (S1)$$

$$\text{Porosity }(\%)=\frac{\pi\left(\frac{PS}{2}\right)^{2}}{(PS+P\text{-}P)^{2}}\cdot 100 \qquad (S2)$$

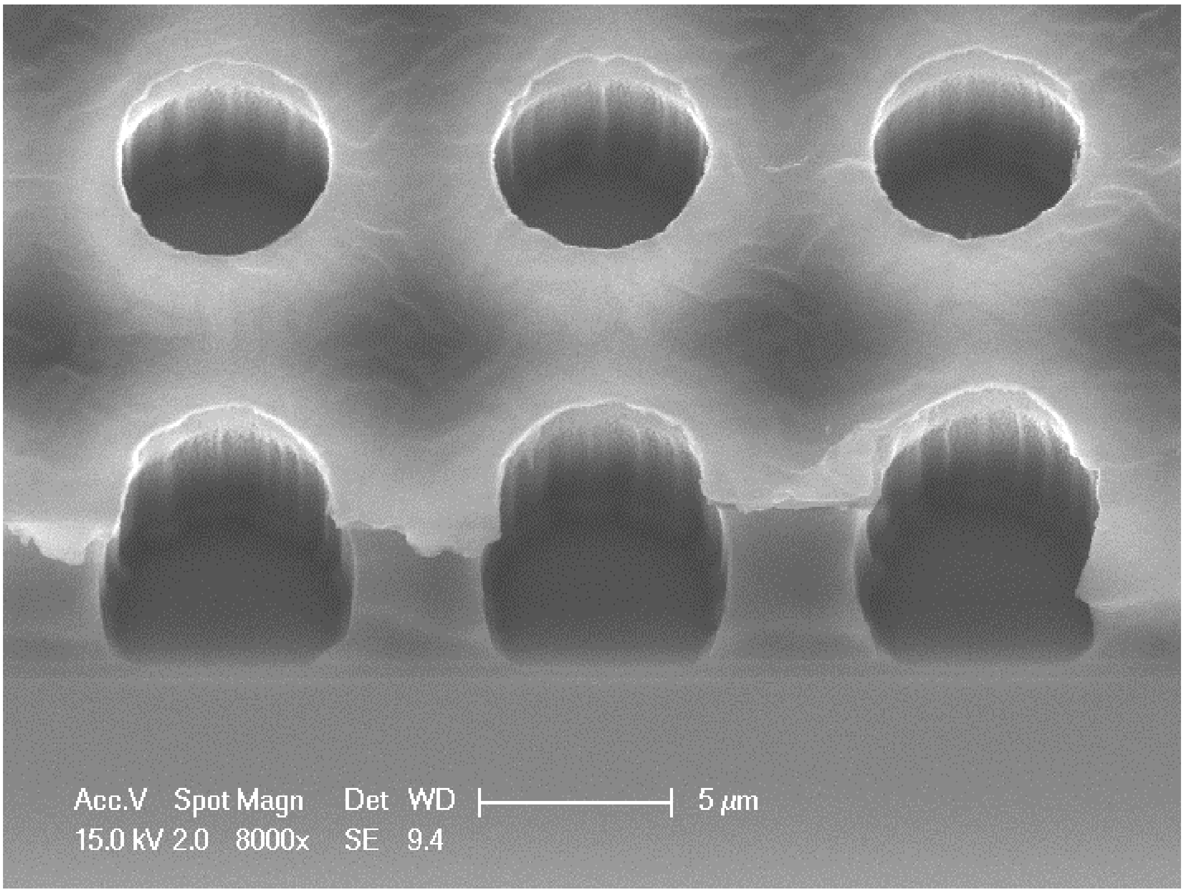

Fig. S1 Cross-section SEM image of the a 8 μm-thick PDMS membrane, with *PS*=5 μm, patterned and transferred with the process here presented. Scale bar: 5μm.

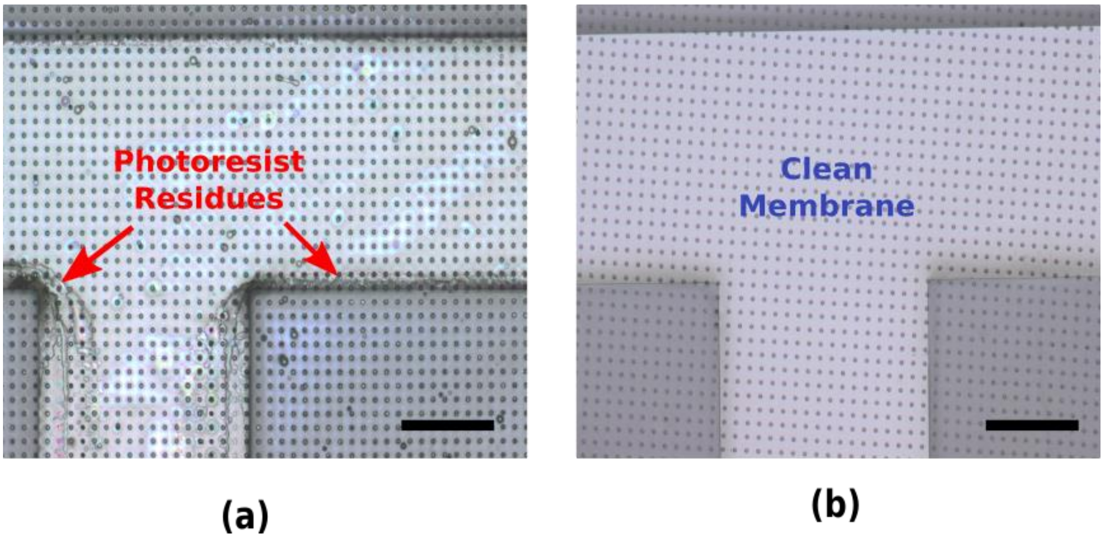

Fig. S2 Optical images of 4 µm-thick PDMS porous membranes after being transferred to an OOC using as sacrificial layer (a) Photoresist and (b) PAA. (a) When using photoresist as sacrificial layer residues are observed on the walls of the microchannel and on the porous membrane surface. (b) After the membrane releasing, no traces of PAA are observed neither to the microchannel walls nor on the porous surface. Scale bars: 200 µm.

2. Additional information on MDA and HUVEC culturing.

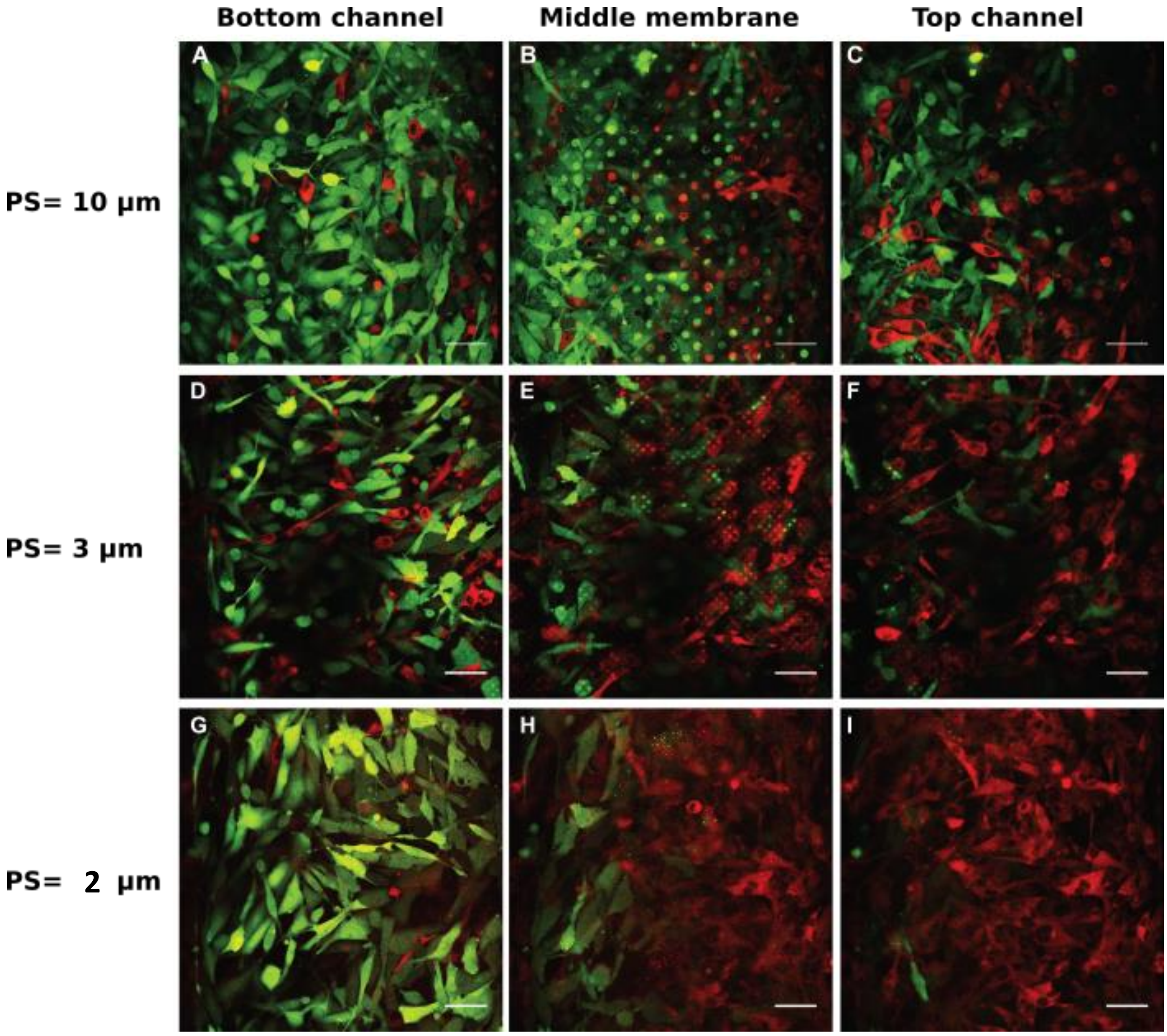

Fig. S3 MDA-cells were transduced with lentivirus either expressing mKO2 with a nuclear export signal (red) or tGFP (green), and were seeded on both sides of the membrane in a parallel channel OOC. Confocal imaging to focus on either the surface of the membrane (B,E,H), top membrane plane (mKO2 cells seeded; C,F,I) or the bottom membrane plane (GFP cells seeded, A,D,G). The cells remained at their side of seeding for PS= 2 μm (G-I) membranes, with sporadic processes protruding through the membrane, recognizable in G by mKO2 positive cell, without the mKO2-negative nucleus.. For PS= 3 μm some exchange of sides could be seen towards the opposite side of the membrane (D-F). For PS= 10 μm, cells could also be identified that had migrated to the opposing side of the membrane, indicating transmigration (Arrows, A-C). Scale bar: 50 μm.

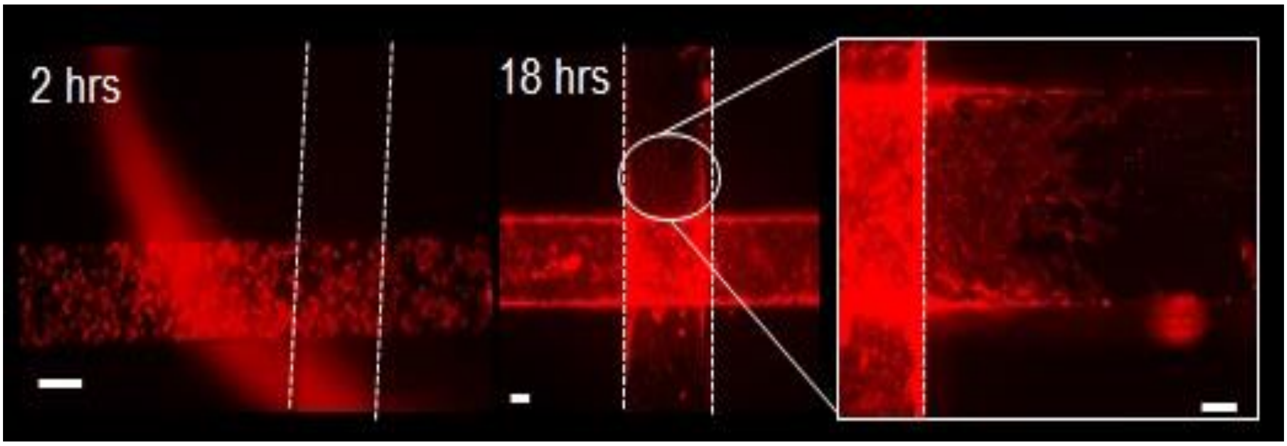

Fig. S4 Cell seeding procedure did not result in cells passing through the membrane. Cell staining was performed after 2 and 18 hours following seeding. At 2 hours after seeding (left), no cells were observed under the membrane whereas after 18 hours (middle), cells can be seen in the adjacent channel (right, inset). Scale bar: 50 µm.

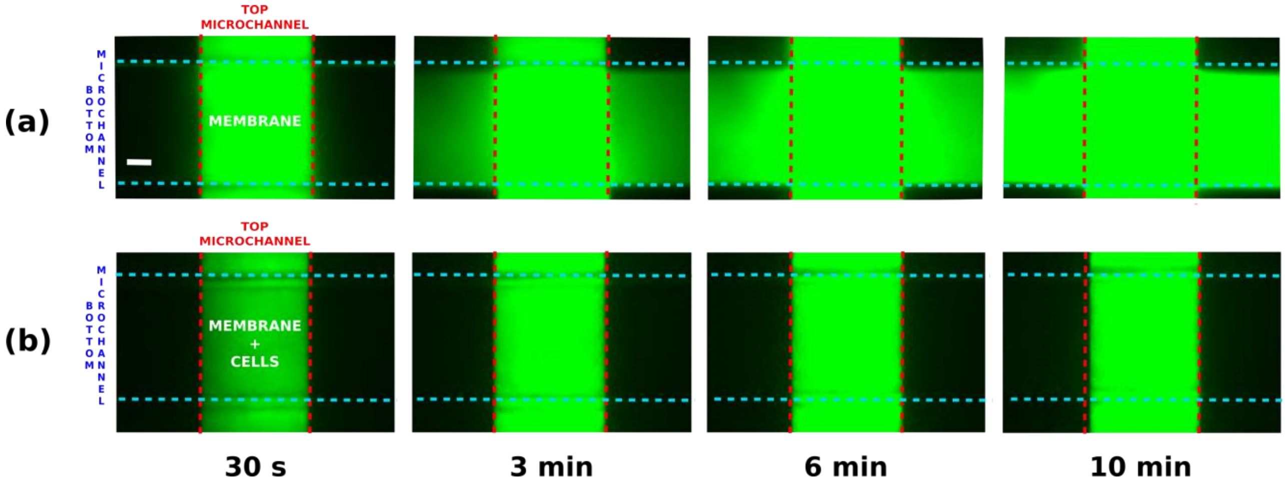

a)

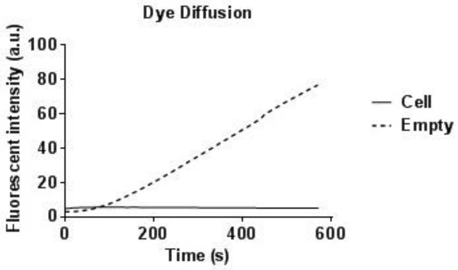

b)

Fig. S5 Barrier function assessment using fluorescein angiography in the OOC with transferred PDMS membranes with and without HUVEC Cells. (a) Following fluorescein (0.3 kDa) administration to the top microchannel (vertical lane, red lines), diffusion of fluorescein to the bottom channel (horizontal lane, blue lines), was visualized by taking sequential images of OOC for 10 min. In an empty microchannel, diffusion of florescent dye started after 30 s. In the microchannel with HUVEC monolayer, diffusion of fluorescent dye is not observed after 10 min. (b) Dye diffusion over the adjacent channel is quantified, where a striking difference can be seen in membranes with and without cells. Scale bar: 100 µm
